# Guizhi Fuling capsule relieves memory deficits by inhibition of microglial neuroinflammation through blocking JAK2/STAT3 pathway in presenilin1/2 conditional double knockout mice

**DOI:** 10.3389/fimmu.2023.1185570

**Published:** 2023-07-03

**Authors:** Guang Yang, Yuting Tong, Xingyu Wang, Chenyi Zhao, Zongtao Ba, Reaila Ahelijiang, Xinjuan Liu, Waimao Gao, Yan Zhao, Yining Gu, Jianmei Yang, Ying Xu

**Affiliations:** ^1^ Department of Physiology, School of Integrative Medicine, Shanghai University of Traditional Chinese Medicine, Shanghai, China; ^2^ School of Rehabilitation Science, Shanghai University of Traditional Chinese Medicine, Shanghai, China; ^3^ Shanghai Yangzhi Rehabilitation Hospital, Tongji University School of Medicine, Shanghai, China; ^4^ Department of Traditional Chinese Medicine, Shanghai Xuhui District Central Hospital, Shanghai, China

**Keywords:** Guizhi Fuling capsule, Alzheimer’s disease, memory deficits, microglial activation, neuroinflammation, JAK2/STAT3 pathway

## Abstract

Chronic neuroinflammation has been regarded as an important part of the pathological initiation of Alzheimer’s disease (AD), which is associated with the regulation of microglial activation. Preventing microglial activation to inhibit neuroinflammation may become a potential target for the treatment of neurodegenerative diseases. Guizhi Fuling capsule (GZFL) has a strong repression on inflammatory responses. Here, the presenilin1/2 conditional double knockout (PS cDKO) mice, a well-established mouse model of AD, were divided into: WT mice (WT), WT mice+GZFL (WT+GZFL), PS cDKO mice (cDKO), and PS cDKO mice+GZFL (cDKO+GZFL). Mice in the WT+GZFL and cDKO+GZFL group were fed standard chow containing 2000 ppm GZFL for 90 days. After 60 days of GZFL treatment, mice were given to behavioral tests for 30 days in order to explore the effects of GZFL on cognitive and motor function. Then, mice were sacrificed for examining the effects of GZFL on inflammation. Furthermore, primary microglia were obtained from neonatal Sprague-Dawley rats and pretreated with or without GZFL (50 μg/ml) for 1 h in the absence or presence of lipopolysaccharide (LPS) (100 ng/ml) stimulation to speculate whether the underlying mechanism of GZFL’s anti-inflammatory potential was closely associated with Janus kinase 2 (JAK2)/signal transducer and activator of transcription 3 (STAT3) signaling pathway. Our findings indicated that GZFL has the ability to alleviate memory deficits in PS cDKO mice, which attributes to the improvement of neuroinflammation by inhibiting microglial activation and the levels of pro-inflammatory mediators. In addition, GZFL could inverse the tau hyperphosphorylation and the lessened expression of synaptic proteins in hippocampus of PS cDKO mice. Furthermore, GZFL prevented LPS-induced neuroinflammatory responses in primary microglia by decreasing the levels of pro-inflammatory mediators. It is noteworthy that therapeutic effects of GZFL on memory impairment are depended on the inhibition of neuroinflammatory responses by the blockage of JAK2/STAT3 signaling pathway. Taken together, GZFL may be an effective compound Chinese medicine for the improvement and postponement of neurodegenerative progression in AD.

## Highlights

- GZFL ameliorates memory impairment *via* inversing synaptic proteins deficits in presenilin1/2 conditional double knockout mice with AD-like phenotypes.
- GZFL exhibits neuroprotective effects by inhibiting microglial neuroinflammation *via* the blockage of JAK2/STAT3 signaling pathway.
- GZFL symbolizes an effective compound Chinese medicine for the improvement and postponement of neurodegenerative progression in AD.

## Introduction

Alzheimer’s disease (AD) is a common cause of dementia, which has become one of the most urgent public health problems in an era of serious population aging (1). As early as 1907, the pathological features of AD were described by the neuroanatomist Dr. Alois Alzheimer as brain atrophy, β-amyloid (Aβ) deposition, neurofibrillary tangle (NFT) aggregation and neuronal loss (2). Besides these pathological characteristics, AD is generally accompanied by severe neuroinflammation presented as microgliosis, astrogliosis, and the rising levels of pro-inflammatory mediators (3). Although current researchers at home and abroad have conducted unremitting researches on the prevention and treatment of AD, there is still a lack of more effective treatments for AD in view of the complexity of pathological mechanism.

Neuroinflammation and microglial activation are crucial parts in the pathogenesis of neurodegenerative diseases, especially in AD (4). Microglia, as immune sentinels involved in a potent inflammatory response, are responsible for preserving the homeostasis of the microenvironment in the brain (5). Immune responses induced by microglia and macrophages take part in a coordinated pattern to evoke the first line of protection against toxic substances from both internal and external sources (6). In the event of pathological stimulation, tissue destruction or imbalance of homeostasis, microglia respond by the alteration of morphology, antigen presentation, phagocytosis and secretory activity in order to maintain the integrity of central nervous system (CNS) (7). In these situations, microglial activation is induced by pro-inflammatory or anti-inflammatory molecules, manifesting as damage-associated and pathogen-associated molecular patterns (DAMPs-PAMPs) (8). The early release of pro-inflammatory mediators is conducive to neural plasticity, brain homeostasis and plaque clearance (9). However, long-term sustained pro-inflammatory or neurotoxic mediators secreted from activated microglia owing to chronic neuroinflammation such as nitric oxide (NO) and cytokines have a central influence in producing toxicity to neurons and making the damage to synaptic plasticity, ultimately leading to the degeneration of neurons and deficits of learning and memory ability, accompanied by the increasing occurrence of neurodegenerative diseases, such as AD (10). Therefore, alleviating neuroinflammation *via* inhibiting microglial activation could be a potential therapeutic target for the treatment of AD.

Currently, the existing AD drugs can only temporarily improve memory impairment, however, they are unable to completely cure AD (11). The potential advantage of traditional Chinese medicine compound is their composition of various active components, which can carry out comprehensive treatment for diseases *via* multi-component, multi-target and multi-pathway. Guizhi Fuling capsule (GZFL), consisting of five Chinese medicinal materials such as Cinnamomi Ramulus, Paeoniae Radix Alba, Moutan Cortex, Persicae Semen, and Poria, is firstly described by Chinese physician Zhongjing Zhang in the traditional Chinese medical book “*Synopsis of Prescriptions of the Golden Chamber*”. GZFL possesses a variety of pharmacological effects on regulating the endocrine system and renal function, improving the injury after cerebral ischemia-reperfusion, expanding microvessels, and often uses for analgesia, sedation and anti-inflammatory (12). Previous research has reported that GZFL could significantly decrease the expression of pro-inflammatory cytokines including interleukin-1 beta (IL-1β) and tumor necrosis factor alpha (TNF-α), and increase the expression of anti-inflammatory cytokine (IL-10) with its receptor in ischemia-reperfusion model of rat (13). In addition, a metabolomics study has speculated that GZFL might down-regulate the synthesis of arachidonic acid and regulate the expression of cyclooxygenase-2 (COX-2) in order to reduce the release of inflammatory mediators for alleviating dysmenorrhea (14). These results consistently suggested that GZFL has a strong inhibitory effect on inflammation. However, it is unknown whether GZFL is capable of showing the same anti-inflammatory and neuroprotective effects in AD mouse model.

Presenilin (PS) proteins, including PS1 and PS2, are compounded from the γ-secretase complex, which involve in regulating proteolytic processing for amyloid precursor protein and playing a significant role in cell differentiation, gene transcription and disease progression (15). PS1/PS2 conditional double knockout (PS cDKO) mice display a series of age-dependent AD-like phenotypes, including synaptic dysfunction, tau hyperphosphorylation and severe neurodegeneration (16) by 2 months of age and a more severe phenotype by 6 months of age. A significant increase in the expression of pro-inflammatory factors has been observed in PS cDKO mice, accompanied by a trend of peripheral diffusion from the CNS (17). Therefore, 3-month-old mice were selected as our research objects and treated with GZFL (2000 ppm) for consecutive 90 days here. Behavioral and molecular biological methods were used to explore the effects of GZFL on improving memory deficits, inflammatory response, impaired synaptic function and tau hyperphosphorylation. Besides, a cellular inflammation model was established by LPS treatment to further investigate the effects of GZFL on neuroinflammation in LPS-stimulated primary microglia and clarify the underlying mechanism. We found that GZFL was capable of ameliorating memory deficits without locomotor alteration, up-regulating the expression of synaptic protein and down-regulating the hyperphosphorylation of tau protein, probably attributing to the anti-inflammatory effect of GZFL by inhibition of microglial activation in the hippocampus (HPC) of PS cDKO mice. Mechanically, we determined that GZFL suppressed microglia-mediated inflammatory responses partly *via* the blockage of JAK2/STAT3 signaling pathway.

## Materials and methods

### Animals

The generation and genotyping of PS cDKO mice were showed as described previously (16). Mice possessed with the transgene Cre+/−, PS1f/f, and PS2−/− were applied as PS cDKO mice, however, their littermates with the transgene Cre+/−, PS1+/+ and PS2+/+ were used as wild-type (WT) controls. 3-month-old PS cDKO mice and their littermates weighed 25 ± 5 g were chosen in this study with half males and half females. Mice were available to food and water, and were raised in specific pathogen free animal room at a comfortable temperature of 22 ± 2°C in 12-h light-dark cycles (lights on at 7 am). All experimental procedures were operated in accordance with the provisions of animal ethics and approved by the Animal Care and Use Committee of Shanghai University of Traditional Chinese Medicine.

### Drug administration

GZFL capsules composed of Cinnamomi Ramulus, Paeoniae Radix Alba, Moutan Cortex, Persicae Semen, and Poria, from Jiangsu Kangyuan Pharmaceutical Co., Ltd (1506306942, Jiangsu, China). PS cDKO mice and their littermates were randomly assigned to four groups with 20 in each: WT mice (WT), WT mice+GZFL (WT+GZFL), PS cDKO mice (cDKO), and PS cDKO mice+GZFL (cDKO+GZFL). The mice were fed standard chow in the WT and cDKO group. Meanwhile, mice in the WT+GZFL and cDKO+GZFL group were fed standard chow containing 2000 ppm GZFL for 90 days. After 60 days of GZFL treatment, mice were given to behavioral tests for 30 days with continuous administration of GZFL.

### Behavioral tests

**Figure 1:**
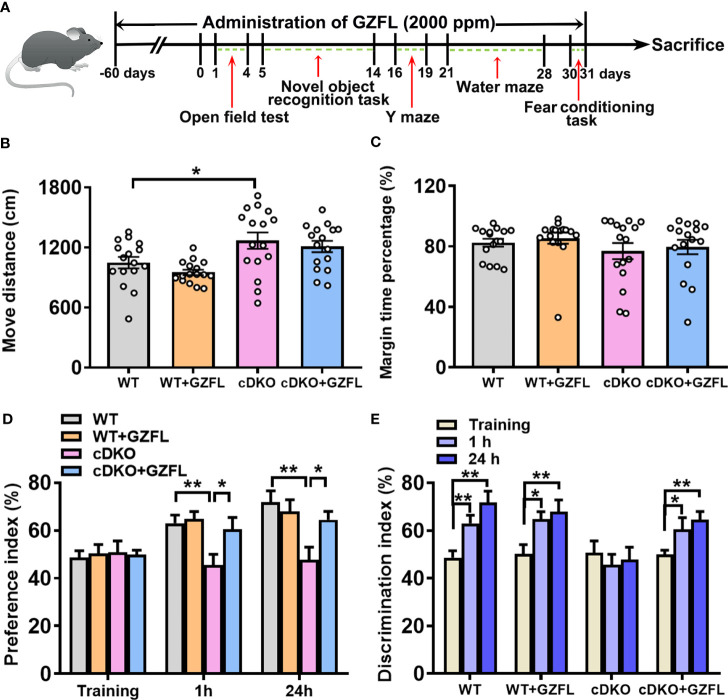
Guizhi Fuling capsule (GZFL) improves recognition memory impairment in presenilin1/2 conditional double knockout (PS cDKO) mice without locomotor alteration. **(A)** The experimental timeline of GZFL treatment and behavioral tests. **(B, C)** An open field test was used to detect the moving distances **(B)** and percentage of time spent in the margin zone **(C)**. **(D)** During novel object recognition task, preference indexes were measured. **(E)** Preference indexes were in comparison in each session within the different groups. Data are the mean ± SEM (n = 16), *P < 0.05; **P < 0.01.

In order to explore the effects of GZFL on cognitive and motor function, the following behavioral tests were carried out in order: open field test, new object recognition task, Y maze, Morris water maze (MWM) and fear conditioning task. Before each behavioral test, the mice were placed in the sound-proofed behavior room for 3 days in advance to adapt to the environment. There were no significant gender differences in the 5 behavioral tests, hence the data of male and female mice were evaluated together. The information and movement tracks of mice among the four groups were obtained by EthoVision XT software (Noldus, Wageningen, The Netherlands). Analysis of the collected data was carried out blinded with respect to study groups. The animal experimental schedule was shown in **Figure 1A**.

#### Open field test

To measure movement ability and anxiety, mice were gently placed in the middle of an open-field box (38 cm × 38 cm × 25 cm) and allowed to move freely for 15 min. Open field image analysis system (Noldus) was used to record the move distances to evaluate locomotor activity. Besides, indexes of anxiety-like behavior were measured by the percentage of time spent in the margin zone. After the test, 75% alcohol was used to wipe the urine and feces in the box and the next experiment was carried out when the odor was completely eliminated.

#### Novel object recognition task

To investigate recognition memory, mice among the four groups were subject to the novel object recognition task. This experiment was divided into two stages, training stage and retention stage. During the training stage, mice were placed in a chamber (40 cm × 40 cm × 25 cm) with two identical objects and permitted to explore the objects for 5 min. 1-h and 24-h after the training stage, one of the objects was substitute for a novel object changed to another appearance. During retention stage, the mice were returned to the same chamber for a 5-min exploration. An index of recognition memory was decided by the recognition ratio (time spent with one of the same objects or the novel one/the total time spent with both objects). Exploratory behaviors of mice were referred to sniffing, touching and biting the objects with the nose, and recorded by applying the tracking system and software (Noldus).

#### Y maze

Mice were subjected to the Y maze in order to evaluate spatial recognition memory. Y maze was constituted by three identical arms (30 cm × 6 cm × 15 cm) arranged equal angles separately around a central connection area. During the training period, the novel arm was shielded by a removable plastic sheet. Mice were gently placed at the start arm and allowed to freely explore the maze for 8 min. 1-h after the training period, the sheet blocked on the previous closed arm was removed and mice were able to move freely for an another 8-min in the three unrestricted arms. The entries and time spent in the novel arm were used as indexes of the spatial recognition memory.

#### Morris water maze

Mice were evaluated for spatial reference memory in the MWM. The equipment was a 120-cm diameter circular pool with 50-cm high walls, which was filled with opaque white water (21–22°C) to a height of 30 cm. There are four quadrants in the maze: target quadrant, right quadrant, opposite quadrant and left quadrant. The platform located in the center of the target quadrant was 10-cm in diameter and drowned 2-cm under the water. Visual cues were stuck on the walls of the circular pool. The first five days of the MWM were referred to the training stage, and mice were gently placed into the water from different quadrants to find the underwater platform within 90 s. The escape latency to the hidden platform and swimming tracks were measured and analyzed by software (Noldus). Mice successfully searching the platform were left on it for 30 s, while mice were unable to find platform were assigned a 90-s latency and guided to the platform followed by remaining for 30 s. On day 6, the probe test was performed after removing the platform from the pool. Mice were released from the middle of four quadrants, and the percentage of the swimming distance and time, and the velocity were recorded for 90 s. The visible platform test was conducted on day 7, the platform installed a vertical pole with a striking flag was placed on the opposite quadrant. The mice were released from the center of each quadrant and searched for the platform within 90 s to evaluate the visual acuity of mice.

#### Fear conditioning task

Fear conditioning task was used to assess associative memory and carried out in a conditioning shock chamber (20 cm × 20 cm × 30 cm) supplemented with a tone generator and activity monitors (Coulbourn Instruments, Holliston, MA, USA). During the training test, the mice were gently put into the chamber to explore for 3 min. Then, mice were exposed to a 30-s tone (75 dB) followed immediately by a 2-s foot shock at 0.45 mA. After stimulating by conditioning of the tone and shock, the freezing reactions of mice were immediately recorded for 30 s. 24 h latter, mice were returned to the same chamber without the stimulation of sound and electric shock, and the freezing durations were recorded for 4 min. Then, the mice were placed into a novel chamber for two successive 3 min sessions in the presence or absence of a tone stimulus, and their freezing responses showing the pretone freezing and cued freezing were separately recorded. Freezing responses were operationally defined as complete immobility other than respiratory movements. Freezing behavior was analyzed by software and freezing responses were subsequently calculated by experimenters blinded to the groups of mice [(total freezing time/total testing time) × 100%].

### Immunohistochemistry staining and quantitative analysis

For IHC staining, mice brains were stripped and perfused with 4% cold paraformaldehyde overnight at 4 °C followed by transferring into 30% sucrose solution. Brain sections (20 μm) were cut through the dorsal HPC by a freezing microtome (Thermo Scientific™ HM525, Thermo Fisher Scientific, Waltham, MA, USA) and soaked at -20°C in cryoprotectant. After scouring in PBS, the sections were subject to a 10-min incubation with 0.3% hydrogen peroxide (H_2_O_2_) to inhibit endogenous peroxidase activity and then blocked by 10% bovine serum albumin (BSA, Beyotime, Jiangsu, China) at room temperature for 2 h. Subsequently, the sections were incubated with anti-Iba1 polyclonal antibody (1:1000, WAKO Chemicals, Chuo-ku, Osaka, Japan) for 16 h at 4°C. Sections were washed with PBS and treated with a biotinylated secondary antibody (1:200, Invitrogen, Carlsbad, CA, USA) for 2 h followed by an another 2-h incubation with avidin-biotin-peroxidase complex (1:1000, Invitrogen). Chromatic reaction of the brain sections was visualized with 3,3′-diaminobenzidine (DAB, Sigma-Aldrich, St.Louis, MO, USA). After treating with anti-fluorescence quenching reagent (Invitrogen), the brain sections were imaged by using an Axio Imager 2 visible/fluorescence microscope (Carl Zeiss, Oberkochen, Germany).

To quantify the microglial density, the number of Iba1^+^ cells in HPC CA1 at least 5 images (40× objective magnification) per mouse was bilaterally calculated. The ratio of amoeba/activation to total Iba1^+^ microglia in HPC CA1 was quantified on the basis of microglial morphology. Briefly, resting microglia were described as the cell with a small elongated cell body accompanied by radially ramified processes. However, activated microglia were described as the cell with an enlarged circular cell body surrounded by short retracted processes. Image J Skeleton Analysis was used to assess the branching numbers, average, and longest branches lengths of the microglia among the groups of brain sections. 5 brain sections collected from each mouse were averaged to obtain a mean value, and a total of six mice were selected in each group. Image analysis was carried out blinded with respect to the research group.

### Western blot analysis

In order to prepare lysates from the brain tissues of mice in each group, mice were sacrificed after behavioral tests. Subsequently, the brains were carefully stripped and the HPC was dissected. The brain tissues were homogenized in ice-cold radio-immunoprecipitation assay buffer (Beyotime Biotechnology, Jiangsu, China) mixed uniformly with 1 mM phenylmethanesulfonyl fluoride, protease inhibitor and phosphatase inhibitor. To obtain primary microglial lysates, cells planted in 6-well plates (2×10^6^ cells/well) were cultured in the presence or absence of LPS (100 ng/ml) with or without GZFL (50 μg/ml) treatment for varying times and then lysed in the same buffer as the tissues. The supernatant of tissues or cells was obtained by a centrifugation at 12,000 rpm for 10 min at 4°C. Approximately 40-μg proteins extracted by the bicinchoninic acid (BCA) protein quantification assay were subjected to SDS–PAGE gel electrophoresis, and then diverted to nitrocellulose membranes (Amersham Biosciences, Buckinghamshire, United Kingdom). These membranes were incubated with primary antibodies overnight at 4°C after blocking with 5% not-fat milk (Genview, Florida, USA) for 1 h, including mouse anti-COX−2 (1:1000, Santa Cruz Biotechnology, Dallas, Texas, USA), rabbit anti-inducible NOS (1:1000, Cell Signaling Technology, Danvers, MA, USA), rabbit anti-NR2A (1:1000, CST), rabbit anti-NR2B (1:1000, CST), rabbit anti-β-actin (1:1000, CST), mouse anti-PSD-95 (1:1000, Santa Cruz Biotechnology), mouse anti-MAP2 (1:1000, Santa Cruz Biotechnology), mouse anti-SYP (1:5000, Santa Cruz Biotechnology), mouse anti-tau (1:1000, Santa Cruz Biotechnology), mouse anti-p-tau (1:1000, Santa Cruz Biotechnology), rabbit anti-p-JAK2 (1:1000, CST), rabbit anti-p-STAT3 (1:1000, CST), mouse anti-JAK2 (1:1000, Santa Cruz Biotechnology) and mouse anti-STAT3 (1:1000, Santa Cruz Biotechnology). Then, after a thorough washing of the membranes with TBST, the membranes were incubated with secondary antibody (1:3000, CST) at room temperature for 1 h. Subsequently, protein bands were visualized by Image Quant software (Tanon, Shanghai, China) and Image J software was used to quantify the intensities of protein bands. The relative protein levels were normalized to β-actin levels.

### Quantitative real-time PCR

Primary microglia prepared for PCR analysis were stimulated with or without LPS (100 ng/ml) in the presence or absence of GZFL (50 μg/ml) treatment within 6-well plates (2×10^6^ cells/well) at different times. Primary microglia or tissues were dissociated and homogenized with TRIzol reagent (Invitrogen) for RNA isolation. Then, reverse transcription was performed by the Primescript™RT reagent kit with gDNA Eraser (Takara Bio Inc. Otsu, Shiga, Japan) according to the manufacturer’s instructions. A SYBR Green kit (TakaRa, Japan) was used to conduct Quantitative real-time PCR from 1 μl of cDNA template. The quantification of β-actin mRNA was served as a loading control for normalization. Fold changes of mRNA levels compared with the control group or WT group were calculated by using 2^-△△Ct^ method. The primer sequences used in PCR are provided in **Table S1**.

### Enzyme-linked immunosorbent assay

To detect the levels of TNF-α, IL-1β and IL-6, the supernatant of the cells and tissues was homogenized by saline. Then, the lysates were analyzed with ELISA kits as per specification, including Mouse IL-1β/IL-1F2 ELISA Set (P234210, R&D Systems, Minneapolis, MN, USA), Mouse TNF (Mono/Mono) ELISA Set (555268, BD Biosciences, Franklin Lakes, NJ, USA), Mouse IL-6 ELISA Set (9036927, BD Biosciences), Rat IL-1β/IL-1F2 ELISA Set (RLB00, R&D Systems), Rat TNF (Mono/Mono) ELISA Set (560479, BD Biosciences) and Rat IL-6 ELISA Set (550319, BD Biosciences).

### Primary microglial preparation and drug administration

Primary microglia were obtained from neonatal Sprague-Dawley rats (day 0-2) according to our previous studies (18). Briefly, cerebral cortices of the brains were separated and digested in the incubator with 0.125% trypsin for 15 min followed by adding an equal volume of culture medium consisting of Dulbecco’s modified Eagle’s medium (DMEM, HyClone, south Logan, Utah, USA), 10% Fetal Bovine Serum (FBS, Hyclone), 10% horse serum (Gibco, Carlsbad, CA, USA) and 1% penicillin-streptomycin (100 U/mL) (Invitrogen) to terminate the digestion. After centrifugation for 5 min at 1,500 rpm, the supernatant of the mixture was removed and the remaining cells were administered with Earle’s balanced salt solution mixed with deoxyribonuclease (DNase) 4 ml/L, 3.82% MgSO4 30 ml/L, Glucose 2.5 g/L and BSA 3g/L. Primary microglia were gathered by centrifugation for 5 min at 1,500 rpm, and then re-suspended with culture medium followed by seeding in Poly-D-Lysine (50 μg/ml)-coated T-75 flasks. Cells were cultured in an incubator (37°C) with 5% CO_2_. Culture medium was replaced every 3 days after seeding. Subsequently, the mixed cells were gently shaken to isolate and purify the primary microglia between days 7-11.

LPS was obtained from Sigma Aldrich (St.Louis, MO, USA) and dissolved in 0.01M PBS. GZFL for cell-based research was resuspended in dimethyl sulfoxide (DMSO, Sigma-Aldrich). After the purified primary microglia adhere to the wall, they were pretreated with or without GZFL (50 μg/ml) for 1 h in the absence or presence of LPS (100 ng/ml) stimulation. Untreated primary microglia were used as controls.

Ruxolitinib (INCB018424) and C188-9 were obtained from Selleck Chemicals (Houston, TX, USA) and dissolved in DMSO to make stock solution, stored at -20°C. A total of 2×10^6^ cells were seeded in each well of 6-well plates for experiments to explore the mechanism of GZFL on improvement of neuroinflammation. Ruxolitinib, a potent JAK1/JAK2 inhibitor, was used to inhibit the signaling pathway of JAK2 signal transducers and activators of STAT3 (19). Cells were pretreated by GZFL (50 μg/ml) for 1 h and then exposed to LPS (100 ng/ml) with or without 5 μM Ruxolitinib for 24 h. C188-9, a small molecular inhibitor, was used for suppressing STAT3 activation (20). Cells were administered to the indicated concentration of C188-9 (10 μM) and cultured for 12 h at 37°C.

### Cell viability assay

3-(4,5-dimethylthiazol-2-yl)-2,5-diphenyltetrazolium bromide (MTT) reduction assay was used to detect the viability of primary microglia. Primary microglia were planted in 96-well plates (2×10^4^ cells/well) and then treated with varying concentration of GZFL (12.5-400 μg/mL) for 24 h. After a 4-h incubation with 20 μl MTT staining solution (5 mg/ml in PBS), 200 μl supernatant was sucked away per well and the precipitated MTT methylzan crystal was dissolved with 150 μl DMSO. The absolution value was detected by a microplate reader (Synergy 2, BioTek Instruments, Inc, Winooski, VT, USA) at 570 nm and outcomes were indicated by the percentage of microglia in groups treated with GZFL over the untreated group. Each assay was repeated in triplicate.

### Nitrite quantification

The amount of nitrite was measured to evaluate NO production which was secreted from activated microglia. Primary microglia used for nitrite quantification were cultivated in 24-well plates (2×10^5^ cell/well) and subjected to varying concentration of GZFL (6.25-400 μg/mL) treatment with LPS (100 ng/ml). Previous studies have reported that the accumulation of nitrite in microglial culture medium was evaluated by Griess reaction (21). 100 μl Griess reagent, composing of 1% sulfonamide water and 0.1% N-1-naphthalene ethylenediamine hydrochloride in 5% phosphoric acid, was mixed with equal volume of the conditioned medium per well and the absorbance of reaction mixture was measured at 540 nm. Each assay was repeated in triplicate.

### Statistical analysis

All data are presented as the mean ± SEM, and p<0.05 was indicated as a significance level. The SPSS software (IBM-Version-24) was used to analyze the data by using a one-way, two-way, or repeated measurement ANOVA with Bonferroni *post hoc* analyses. In the process of data analysis, the experimenter was blind to the experimental conditions of each mouse.

## Results

### GZFL ameliorates recognition memory impairment in PS cDKO mice without locomotor alteration

In line with previous studies, PS cDKO mice were severely impaired in memory and behavior at six months of age (16). Firstly, an open field experiment was used to evaluate the spontaneous locomotor activity of PS cDKO mice. We observed an increase in the movement distance of PS cDKO mice compared with WT mice (**Figure 1B**). However, GZFL treatment did not change the movement distance and marginal zone time percentage in either WT or PS cDKO mice (**Figures 1B, C**), which indicated that GZFL had no influence on the spontaneous locomotor ability of PS cDKO mice.

Then, mice were subject to the novel object recognition test to explore whether GZFL has a therapeutic effect on recognition memory deficits of PS cDKO mice. In the training stage, the results of inter group comparison showed that there was no obvious preference between the novel object and the familiar object among each group (**Figure 1D**). After training for 1 h (for short-time memory) and 24 h (for long-time memory), PS DKO mice spent less time exploring novel object compared to WT mice, which implied an impairment of recognition memory in PS DKO mice. Notably, the administration of GZFL had a beneficial effect on short-term memory and long-time memory, which could promote mice to show a preference for the novel object rather than the familiar object (**Figure 1D**). Moreover, on the basis of intra group comparison, mice in all groups showed a significantly higher preference for novel object except for PS cDKO group. During the 1-h and 24-h test phases, we found that PS DKO mice treated with GZFL spent more time on the novel object (**Figure 1E**), suggesting that GZFL had the ability to improve impaired short-term and long-term recognition memory.

### GZFL moderates spatial and associative memory impairment in PS cDKO mice

**Figure 2:**
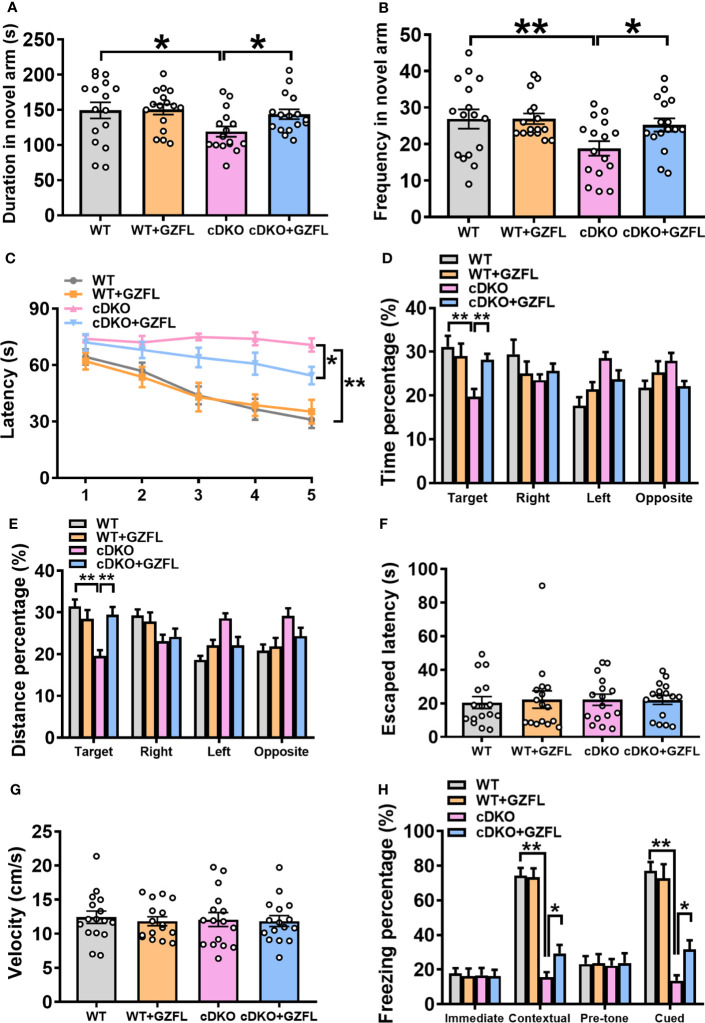
Guizhi Fuling capsule (GZFL) relieves spatial and associative memory impairment in presenilin1/2 conditional double knockout (PS cDKO) mice. **(A, B)** Duration **(A)** and frequency **(B)** entered into the novel arm of Y maze. **(C)** The escape latency to find the hidden platform during the first 5-d training period of Morris water maze (MWM). **(D, E)** During the probe phase of MWM, the percentage of time **(D)** and distance **(E)** were tested in the target quadrant. **(F)**The escape latency to seek out the visible platform in MWM. **(G)** The swimming speed during the test period of MWM. **(H)** The freezing response in the fear conditioning task. Data are the mean ± SEM (n = 16), *P < 0.05; **P < 0.01.

The spatial memory and associative memory of PS cDKO mice aged 2-6 months were deteriorated significantly, which has been deeply investigated (22). Thus, Y maze and MWM were selected to further explore the effects of GZFL on spatial memory in PS cDKO mice. Mice naturally have the characteristics of exploring new environments, therefore, it was consistent with the previous research results that WT mice showed a preference for the novel arm in Y maze (23). Compared to WT mice, PS cDKO mice spent less time exploring the novel arm and they rarely visited the novel arm (**Figures 2A, B**), undoubtedly indicating that PS cDKO mice had serious spatial recognition impairment. However, PS cDKO mice treated with GZFL displayed an increase in the duration spent and frequencies entered into the novel arm (**Figures 2A, B**), which demonstrated that GZFL had a great advantage in improving the impaired spatial recognition memory in PS cDKO mice.

On the first day of the place navigation test in MWM, there was no significant differences in the escape latency to seek out the fixed underwater platform among the four groups (**Figure 2C**). In the next 4-day training session, PS cDKO mice spent more time finding the hidden platform than WT mice, which indicated that it was difficult for them to find the hidden platform. However, it markedly took less time for PS cDKO mice receiving GZFL treatment to find the hidden platform (**Figure 2C**). After removing the underwater platform, a spatial probe test was used to test the retention memory of the mice on day 6. The results showed that PS cDKO mice swam a shorter distance and spent less time in the target quadrant. However, administering GZFL to PS DKO mice significantly increased the occupancy of distance and time in the target quadrant (**Figures 2D, E**). It was worth noting that the swimming speed during the test were indistinguishable among four groups of mice (**Figure 2G**). No significant differences were discovered on the ability to find platform during the visible platform task on day 7 (**Figure 2F**), illustrating that mice in each group showed similar levels of normal sensorimotor abilities. In brief, GZFL could relieve the spatial reference memory deficits in PS DKO mice.

Further, fear conditioning task was used to probe whether GZFL could alleviate the associative memory impairment in PS cDKO mice. During the training phase, the immediate freezing of mice in each group were at a low level. During the retention test, the levels of contextual and cued freezing were remarkably reduced in PS DKO mice compared to WT mice, showing a poor performance of associative memory in PS cDKO mice. However, PS cDKO mice receiving GZFL treatment performed significantly higher levels of contextual and cued freezing (**Figure 2H**). In conclusion, the above-mentioned results suggested that GZFL had a practical drug effect on improving both the damaged spatial memory and associative memory of PS cDKO mice.

### GZFL inhibits microglial activation in HPC CA1 region of PS cDKO mice

**Figure 3:**
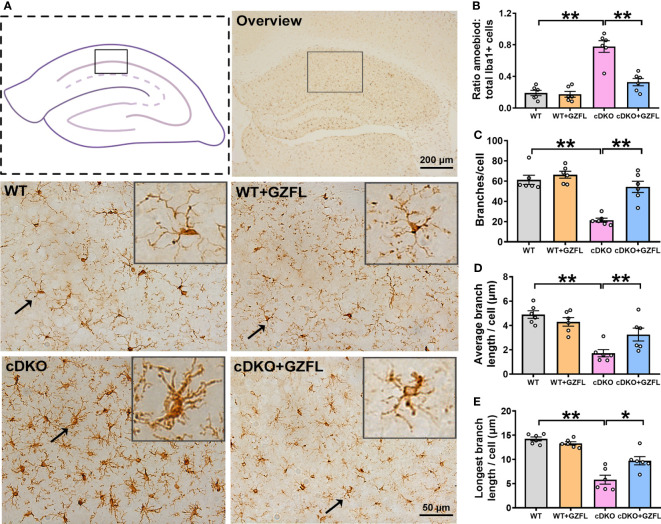
Guizhi Fuling capsule (GZFL) represses microglial activation in the hippocampus (HPC) CA1 of presenilin1/2 conditional double knockout (PS cDKO) mice. **(A)** Representative images of Iba1^+^ microglia in HPC CA1. The location of HPC CA1 was displayed in overview images by the box. Magnification of overview image is 5×, scale bar, 200 μm. Magnification of other four images is 40×, Scale bar, 50 μm. **(B)** The percentage of amoeboid cells in total Iba1^+^ cells were calculated to conduct a quantitative analysis of microglial activation in HPC CA1. **(C–E)** The branches **(C)**, the average branch length **(D)** and longest branch length **(E)** in HPC CA1. Data are the mean ± SEM (n = 6), *P < 0.05; **P < 0.01.

Microglia are important contributors to neurodevelopment, neuroinflammation and neurodegeneration (24), which play a key role in the innate immune response of neurodegenerative diseases, especially in AD (25). In order to explore whether GZFL could inhibit neuroinflammation mediated by microglial activation, we stained Iba1 protein, a specific marker for microglia, to observe the morphologic alterations of microglia in HPC CA1 region of the mice between each group. DAB immunostaining showed that microglia were under the situation of static with small cell bodies and slender branches in HPC CA1 of WT mice. Oppositely, PS cDKO mice showed higher proportion of amoeboid/activated microglia in HPC CA1, which had more rounded cell bodies accompanied by shorter retracted processes. However, after GZFL treatment, the morphology of microglia switched to a resting state with smaller cell bodies around more protruding branches (**Figure 3A**). Furthermore, the results showed that PS cDKO mice emerged an increased proportion of amoeboid in total microglia with lower branching numbers, and both the average length and the longest branch length were shortened (**Figures 3B–E**). Surprisingly, administering GZFL to PS cDKO mice could reverse these manifestations, suggesting that the improvement of memory deficits after GZFL treatment might depend on the inhibition of microglial activation in HPC CA1 of PS cDKO mice.

### GZFL diminishes the increased levels of pro-inflammatory mediators in the HPC of PS cDKO mice

**Figure 4:**
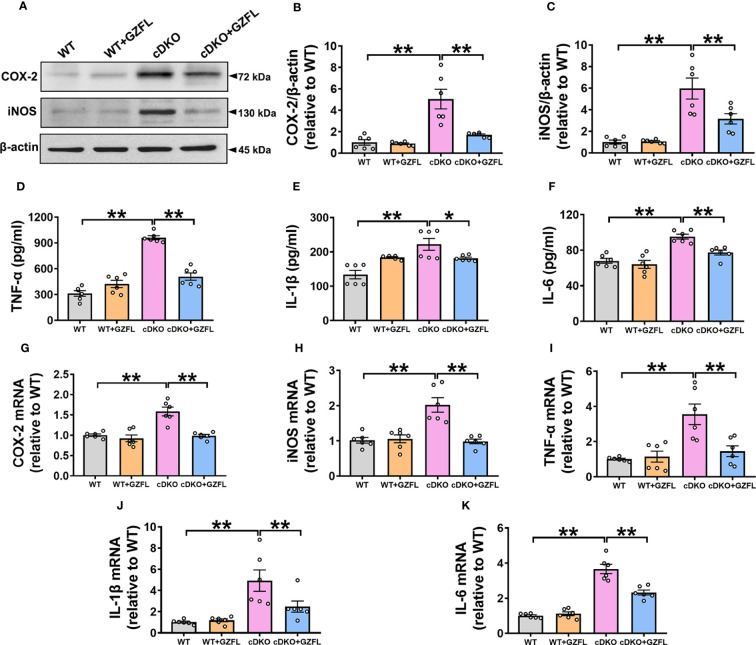
Guizhi Fuling capsule (GZFL) decreases the increased levels of pro-inflammatory mediators in the hippocampus (HPC) of presenilin1/2 conditional double knockout (PS cDKO) mice. **(A)** Representative Western blot for COX-2 and iNOS in HPC. **(B, C)** Quantification of Western blot for COX-2 and iNOS in HPC. **(D–F)** The levels of TNF-α **(D)**, IL-1β **(E)** and IL-6 **(F)** were measured by ELISA in HPC. **(G–K)** The mRNA levels of COX-2 **(G)**, iNOS **(H)**, TNF-α **(I)**, IL-1β **(J)** and IL-6 **(K)** in HPC were analyzed by qRT-PCR. Uncropped immunoblots are shown in **Supplementary Figure 1**. These values were showed as relative changes to the respective wild-type (WT) mice, which was set to 1. Data are the mean ± SEM (n = 6), *P < 0.05; **P < 0.01.

Neuroinflammation is an important mechanism leading to the progressive degeneration of nerve cells (26). One of the characteristics of neuroinflammation is microglial activation, which produces pro-inflammatory mediators and causes neuronal damage, eventually resulting in the deterioration of AD (27). Therefore, the levels of pro-inflammatory mediators in HPC of mice such as iNOS, COX-2, IL-1β, TNF-α and IL-6 were measured to investigate the effects of GZFL on neuroinflammation. Western blot and ELISA analysis were utilized to detect the protein levels of pro-inflammatory mediators in HPC of mice among the four groups. Our data showed a notable increase in the levels of iNOS, COX-2, IL-1β, TNF-α and IL-6 in HPC of PS cDKO mice, while GZFL treatment could reverse the elevated protein levels of these pro-inflammatory mediators (**Figures 4A–F**).

Furthermore, qRT-PCR was performed to evaluate the mRNA levels of pro-inflammatory mediators. The results showed that the mRNA levels of iNOS, COX-2, IL-1β, TNF-α and IL-6 were remarkably up-regulated in PS cDKO mice. Similarly, we found that the increased mRNA levels of iNOS, COX-2, IL-1β, TNF-α and IL-6 were prevented by GZFL administration (**Figures 4G–K**). Together, these observations suggested that GZFL could play a key role in inhibiting neuroinflammatory responses by reducing the expressions of pro-inflammatory mediators in HPC of PS cDKO mice.

### GZFL increases the expressions of synaptic proteins and decreases tau hyperphosphorylation in HPC of PS cDKO mice

**Figure 5:**
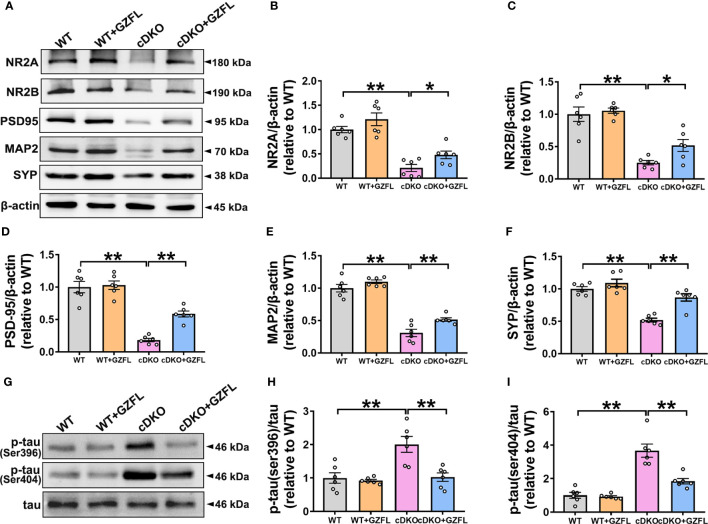
Guizhi Fuling capsule (GZFL) raises the expressions of synaptic proteins and decreases the phosphorylation of tau protein in the hippocampus (HPC) of presenilin1/2 conditional double knockout (PS cDKO) mice. The brain tissues of mice in each group were lysed, quantified and then analyzed by Western blot to detect the expressions of synaptic proteins (NR2A, NR2B, PSD95, MAP2 and SYP) and phosphorylated tau protein (ser396/404). **(A)** Representative Western blot for synaptic proteins in HPC. **(B–F)** Quantification of Western blot for synaptic proteins in HPC. **(G)** Representative Western blot for phosphorylated tau proteins in HPC. **(H, I)** Quantification of Western blot for phosphorylated tau proteins in HPC. The protein expressions of synaptic proteins and phosphorylated tau proteins were standardized based on the respective expressions of β-actin or total tau. Uncropped immunoblots are shown in **Supplementary Figure 2**. These values were showed as relative changes to the respective wild-type (WT) mice, which was set to 1. Data are the mean ± SEM (n = 6), *P < 0.05; **P < 0.01.

Synaptic plasticity of HPC is closely related to memory ability (28). The pro-inflammatory mediators gradually released by microglial activation are able to cause neuronal damage by destroying synaptic proteins, finally leading to neuronal apoptosis and memory impairment (29). Thus, Western blot was used to detect the N-methyl-D-aspartic acid receptors (NMDAR) including NMDAR 2A subunits (NR2A) and NMDAR 2B subunits (NR2B) and synapse-related proteins, such as postsynaptic density protein 95 (PSD95), synaptophysin (SYP) and microtubule-associated protein 2 (MAP2). The results showed that a significant reduction in the expressions of NR2A, NR2B, MAP2, PSD95 and SYP was observed in HPC of PS cDKO mice, indicating that the structure and function of synapse proteins were seriously destroyed. Surprisingly, the intervention of GZFL could prevent the decrease of the expressions of NR2A, NR2B, MAP2, PSD95 and SYP in HPC of PS cDKO mice (**Figures 5A–F**). These results supported that GZFL had the capability for PS cDKO mice to ameliorate the impaired synaptic structure and function, thereby improving the memory deficits.

One of the typical pathological features of AD is NFT (30). As a microtubule associated protein distributed on the axons of neurons, tau protein is generally abnormally phosphorylated at the sites of ser396 and ser404, which increases the possibility of protein misfolding and eventually leads to NFTs (31). Therefore, for the sake of plumbing the possible mechanism of GZFL on memory amelioration, the expressions of phosphorylated tau proteins were detected among the four groups of mice. Western blot analysis showed that PS cDKO mice manifested significantly elevated expressions of tau phosphorylation at ser396 and ser404 in comparison with WT mice in HPC. Intriguingly, administering GZFL to PS cDKO mice led to a discernible reduction in the expressions of tau hyperphosphorylation in HPC of PS cDKO mice (**Figures 5G–I**). The earlier mentioned findings supported a possibility that the improvement of GZFL on memory impairment was due to the inhibition of tau hyperphosphorylation.

### GZFL reduces the levels of pro-inflammatory mediators in LPS-stimulated primary microglia without cytotoxicity

**Figure 6:**
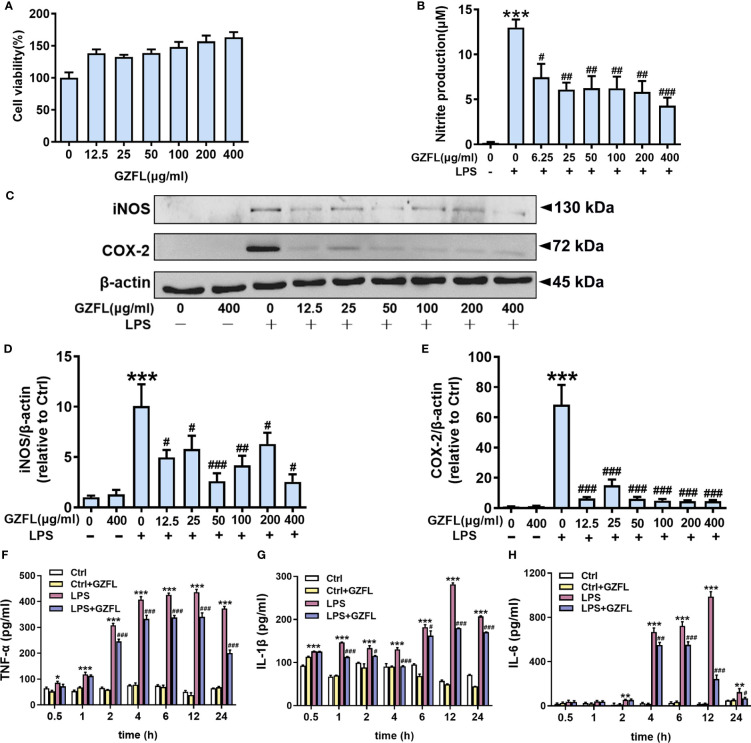
Influences of Guizhi Fuling capsule (GZFL) on cell viability, and the levels of pro-inflammatory mediators in primary microglia stimulated by LPS. **(A)** Primary microglia were treated with GZFL (12.5-400 μg/ml) for 24 h followed by MTT assay to measure cell viability. **(B)** Primary microglia were stimulated with LPS (100 ng/ml) with or without GZFL at the indicated concentration (6.25-400 μg/ml) for 24 h followed by Griess assay to detect the level of nitrite in medium. **(C)** Representative Western blot for iNOS and COX-2 in primary microglia pretreated with GZFL (12.5-400 μg/ml) followed by the stimulation of LPS (100 ng/ml). **(D, E)** Quantification of Western blot for iNOS and COX-2 in primary microglia. **(F–H)** Cell media were gathered to analyze the levels of TNF-α, IL-1β and IL-6 by ELISA. The protein expressions of iNOS and COX-2 were standardized based on the respective expressions of β-actin. Uncropped immunoblots are shown in **Supplementary Figure 3**. These values were showed as relative changes to the respective Control, which was set to 1. Data are the mean ± SEM from three independent experiments, *P < 0.05, **P < 0.01, ***P < 0.001 vs untreated Controls; ^#^P < 0.05, ^##^P < 0.01, ^###^P < 0.001 vs LPS alone.

Neuroinflammation has the ability to trigger and promote AD pathology, which is closely related to the activation of microglia (24). Given the earlier mentioned observations of significant impacts for GZFL on memory deficits, we next cultured primary microglia to further clarify the underlying mechanism how GZFL made contributions to neuroprotection. On account of high sensitivity and good repeatability, MTT assay was selected to detect microglial activity. We treated primary microglia with different concentrations of GZFL (12.5-400 μg/ml) for 24 h to detect the cytotoxic effects of GZFL on cells. Our data showed that GZFL at various concentrations did not elicit any difference in cell viability, indicating that GZFL had no harmful effects on microglia. Strikingly, we found that GZFL had the ability to promote the proliferation of microglia (**Figure 6A**).

Due to its neurotoxic effect, NO is often used as an indicator of neuroinflammatory responses and examined by Griess assay. After 24-h treatment of primary microglia stimulated by LPS (100 ng/ml), there was a dramatic increase in the level of nitrite compared to the control group in the cell culture medium. As expected, this up-regulation could be dose-dependently reversed by GZFL (6.25-400 μg/ml) treatment (**Figure 6B**). The above results provided consolidated evidence that GZFL at the concentration between 6.25 μg/ml-400 μg/ml had the capability to safely prevent LPS-induced neuroinflammatory responses in primary microglia.

To examine the role of GZFL at diverse concentrations in ameliorating neuroinflammation, we subsequently detected the expressions of COX-2 and iNOS in primary microglia stimulated with LPS (100 ng/ml) by Western blot. In agreement with previous studies, pro-inflammatory mediators were rarely secreted in resting microglia, while the elevated expressions of COX-2 and iNOS were observed in LPS-stimulated primary microglia (18). To our surprise, the expression of COX-2 was greatly reduced in LPS-induced primary microglia by pretreatment of GZFL (12.5-400 μg/ml). Similar results were obtained in the detection of iNOS expression. Strikingly, the expression of iNOS significantly reduced in LPS-stimulated primary microglia treated by GZFL (50 μg/ml) (**Figures 6C–E**). Together, these findings demonstrated that GZFL (50 μg/ml) could excellently inhibit the expression of iNOS and COX-2 in activated microglia. Hence, GZFL at a concentration of 50 μg/ml was selected as the optimum concentration for the follow-up experiments.

Furthermore, our data showed that the levels of TNF-α and IL-1β were significantly increased in primary microglia stimulated with varied duration of LPS with a peak at 12 h by ELISA assay (**Figures 6F, G**). Similar results were also observed in the level of IL-6, nevertheless, it was 2 h after LPS stimulation that a significant increase could be emerged in the primary microglia (**Figure 6H**). Administering GZFL to primary microglia greatly reduced the level of IL-1β, TNF-α and IL-6 12 h-post LPS stimulation, in spite of the different initial time point token effect by GZFL.

### GZFL suppresses the mRNA expressions of pro-inflammatory mediators in LPS-stimulated primary microglia with obviously morphological characteristics

**Figure 7:**
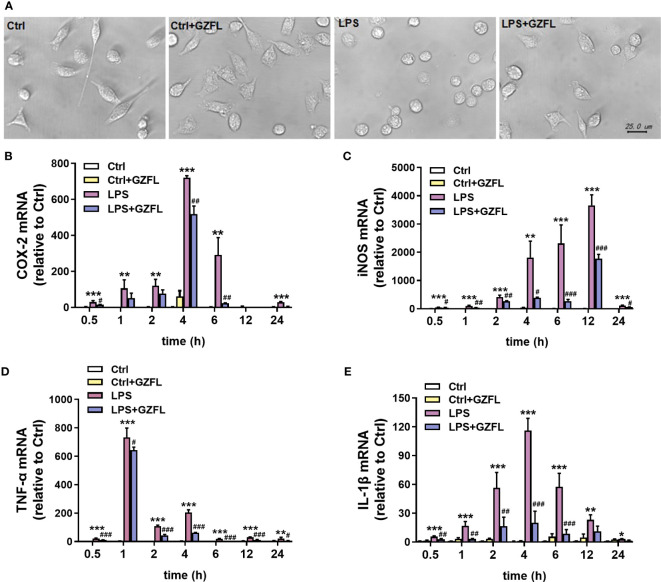
Guizhi Fuling capsule (GZFL) inhibits the mRNA expressions of pro-inflammatory mediators in LPS-stimulated primary microglia with morphological changes. Primary microglia were stimulated by LPS (100 ng/mL) in the absence or presence of GZFL (50 μg/ml) for varying times. **(A)** Morphological changes of primary microglia were observed under a phase-contrast light microscope. Magnification is 40×, Scale bar, 25 μm. Control (Ctrl): untreated primary microglia. Ctrl+GZFL: GZFL (50 μg/ml) treatment alone. LPS: LPS (100 ng/ml) stimulation alone. LPS+GZFL: combined treatment of LPS (100 ng/ml) and GZFL (50 μg/ml). **(C–E)** Total RNA was extracted to quantitate the mRNA expressions of COX-2 **(B)**, iNOS **(C)**, TNF-a **(D)** and IL-1β **(E)** by qRT-PCR. These values are expressed as fold change over the respective Controls. Data are the mean ± SEM from three independent experiments, *P < 0.05, **P < 0.01, ***P < 0.001 vs untreated Controls; ^#^P < 0.05, ^##^P < 0.01, ^###^P < 0.001 vs LPS alone.

To confirm whether GZFL had impacts on microglia exposed to LPS, light microscopy, an instrument vulnerable to observe the morphological characteristics, was used to evaluate the repression of GZFL. The results showed that resting microglia displayed slender cell bodies with branches, while primary microglia stimulated by LPS exhibited larger spherical cell bodies surrounded by retracted synapses. Strikingly, after the pretreatment of GZFL, short branches could be seen in LPS-stimulated primary microglia (**Figure 7A**), indicating that GZFL was able to recover microglial activation to some extent.

The previous research showed that pro-inflammatory mediators, such as IL-1β, TNF-α, COX-2 and iNOS, are voluminously secreted by activated microglia (18). In order to clarify the molecular mechanism that GZFL had the potential to inhibit production of pro-inflammatory mediators induced by LPS, the mRNA levels of pro-inflammatory factors were investigated in LPS-stimulated primary microglia with or without GZFL under different LPS stimulation duration. QRT-PCR showed that the mRNA levels of COX-2 and IL-1β peaked at 4 h, which could be reversed by GZFL treatment in LPS-stimulated primary microglia (**Figures 7B, E**). LPS led to a dramatic raise of the mRNA levels of iNOS and TNF-α in primary microglia stimulated by LPS for different duration. Surprisingly, we found that the mRNA levels of these pro-inflammatory mediators were significantly lower in the LPS-stimulated primary microglia treated by GZFL (**Figures 7C, D**). Together, these findings demonstrated that GZFL could down-regulate the mRNA levels of the pro-inflammatory mediators by various extent.

### GZFL interferes with the JAK2/STAT3 signaling pathway in LPS-stimulated primary microglia

**Figure 8:**
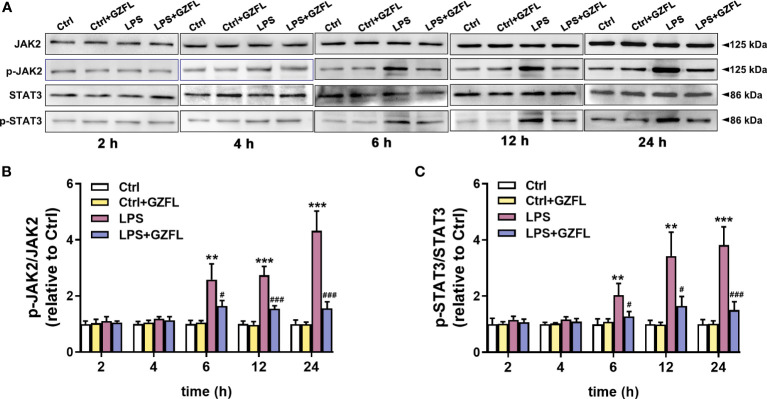
Guizhi Fuling capsule (GZFL) blocks the JAK2/STAT3 signaling pathway in LPS-stimulated primary microglia. Primary microglia were pretreated with GZFL (50 μg/ml) for 1 h followed by the stimulation of LPS (100 ng/ml) for varying times. Microglia were lysed, and the total protein expression of JAK2, STAT3, and their respective phosphorylated forms were assayed by Western blot. **(A)** Representative Western blot for the expressions of JAK2, STAT3, and their respective phosphorylated levels at 2 h, 4 h, 6 h, 12 h and 24 h of LPS stimulation. **(B, C)** Quantification of Western blot for phosphorylated JAK2 **(B)** and STAT3 **(C)**. The expressions of phosphorylated JAK2 and STAT3 were standardized based on the respective total JAK2 and STAT3. Uncropped immunoblots are shown in **Supplementary Figure 4**. These values were showed as relative changes to the respective Control, which was set to 1. Data are the mean ± SEM from three independent experiments, **P < 0.01, ***P < 0.001 vs untreated Controls vs untreated Controls; ^#^P < 0.05, ^###^P < 0.001 vs LPS alone.

The above experiments confirmed that GZFL could inhibit the expression of pro-inflammatory mediators in LPS-stimulated microglia, therefore, we speculated that the underlying mechanism of GZFL’s anti-inflammatory potential was closely associated with neuroinflammation-related pathways. To confirm this hypothesis, we explored the effects of GZFL on JAK2/STAT3 signaling by Western blot with antibodies specifically detecting the phosphorylation levels of JAK2 and STAT3. In the first 4 h of stimulation period, no significant differences in the expressions of JAK2 and STAT3 phosphorylation were observed in primary microglia with LPS treatment in the absence or presence of GZFL. Of note, LPS led to a dramatic increase in the expressions of phosphor-JAK2 and phosphor-STAT3 in primary microglia after 6 h of stimulation, indicating that LPS was an effective activator of JAK2/STAT3 signaling pathway. To our surprise, GZFL significantly reduced the phosphorylation levels of JAK2 and STAT3 in primary microglia exposed to LPS for 6 to 24 h (**Figure 8**). In conclusion, these results suggested that GZFL specifically blocked the activation of JAK2/STAT3 signaling pathway, which might be the mechanism responsible for inhibiting neuroinflammatory responses in LPS-stimulated primary microglia.

### GZFL reduces neuroinflammatory reaction in LPS-stimulated primary microglia by intercepting the JAK2/STAT3 signaling pathway

**Figure 9:**
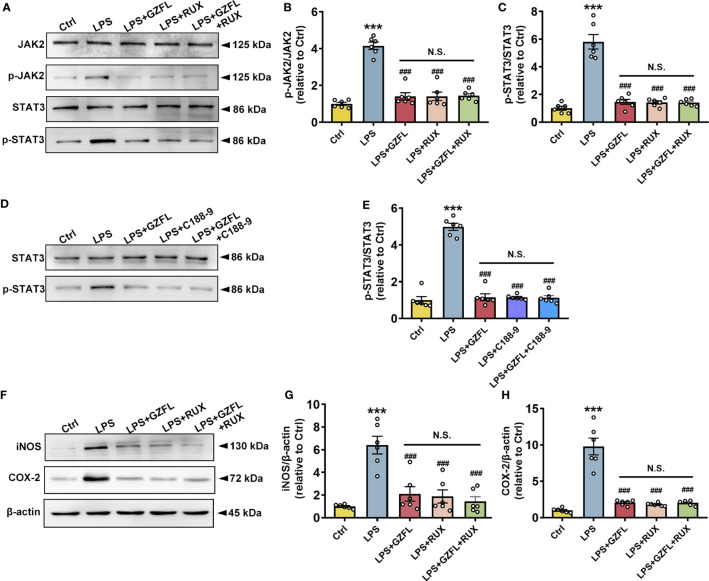
Guizhi Fuling capsule (GZFL) inhibits neuroinflammatory responses in LPS-stimulated primary microglia by intercepting the JAK2/STAT3 signaling pathway. Control (Ctrl): untreated primary microglia. LPS: LPS (100 ng/ml) stimulation alone. LPS+GZFL: combined treatment of LPS (100 ng/ml) and GZFL (50 μg/ml). LPS+Ruxolitinib: combined treatment of LPS (100 ng/ml) and Ruxolitinib (5 μM). LPS+C188-9: combined treatment of LPS (100 ng/ml) and C188-9 (10 μM). LPS+GZFL+Ruxolitinib: combined treatment of LPS (100 ng/ml), GZFL (50 μg/ml) and Ruxolitinib (5 μM). LPS+GZFL+C188-9: combined treatment of LPS (100 ng/ml), GZFL (50 μg/ml) and C188-9 (10 μM). **(A)** Representative Western blot for the total of JAK2, STAT3, and their respective phosphorylated levels. **(B, C)** Quantification of Western blot for phosphorylated JAK2 **(B)** and STAT3 **(C)**. **(D)** Representative Western blot for the total of STAT3 and phosphorylated STAT3. **(E)** Quantification of Western blot for phosphorylated STAT3. **(F)** Representative Western blot for the expressions of iNOS and COX2. **(G, H)** Quantification of Western blot for iNOS **(G)** and COX2 **(H)**. The expressions of phosphorylated JAK2 and STAT3 were standardized based on the respective total of JAK2 and STAT3. The protein expressions of iNOS and COX2 were standardized based on the respective expressions of β-actin. Uncropped immunoblots are shown in **Supplementary Figure 5**. These values were showed as relative changes to the respective Control, which was set to 1. Data are the mean ± SEM from three independent experiments, ***P < 0.001 vs untreated Controls; ^###^P < 0.001 vs LPS alone.

To further investigate potential links between neuroinflammation and JAK2/STAT3 signaling pathway, we examined the influences of GZFL on neuroinflammation after blocking JAK2/STAT3 pathway in LPS-stimulated primary microglia with JAK2/STAT3 inhibitors. We observed that Ruxolitinib, a potent and selective inhibitor of JAK2, obviously decreased the phosphorylation of JAK2 and STAT3 (**Figures 9A–C**), and the expressions of COX-2 and iNOS as well (**Figures 9F–H**). Similar results were observed with the intervention of GZFL in primary microglia stimulated by LPS. On account of the same effects on improving neuroinflammation both in GZFL and Ruxolitinib, we were eager to determine whether they act through the identical mechanism. The results showed that combined treatment with GZFL and Ruxolitinib did not further reduce the phosphorylation of JAK2 and STAT3, and no further reduction in the expression of COX2 and iNOS could be observed in LPS-stimulated primary microglia. In addition, after inhibiting the activation of STAT3 by C188-9, our data showed that both GZFL and C188-9 could obviously reduce phosphorylation level of STAT3, and the combination of them had no effects on further decreasing their phosphorylation (**Figure 9E**). Together, our outcomes provided forceful evidences that GZFL suppressed neuroinflammatory responses by blocking the JAK2/STAT3 signaling pathway in LPS-stimulated primary microglia.

## Discussion

Microglia-mediated neuroinflammation is closely related with the onset and progression of AD (24). As pro-inflammatory mediators released by microglia are the main contributors of neuroinflammation in AD (32), inhibiting the neuroinflammatory responses by the suppression of microglial activation may be a better therapeutic strategy for AD. Our above results provided comprehensive evidences that GZFL might serve as an effective compound of traditional Chinese Medicine for ameliorating the impaired cognitive ability and suppressing the exacerbated neuroinflammatory responses in PS cDKO mice with AD-like syndromes. Besides, these benefits might be attributed to the potential of GZFL for reversing the increased pro-inflammatory mediators, the abnormal expression of synaptic proteins and hyperphosphorylated tau protein in HPC of PS cDKO mice. In particular, we also demonstrated that GZFL had the ability to reduce the levels of pro-inflammatory mediators in LPS-induced primary microglia, which were possibly obtained by obstruction of the JAK/STAT3 signaling pathway. On the basis of the results mentioned above, it could be speculated that GZFL had great potential to improve the cognitive impairment of PS cDKO mice by inhibiting neuroinflammation through blockage of JAK2/STAT3 signaling pathway.

The age-related impairment of memory and cognitive function is a significant pathological feature of AD patients (33). In the light of previous studies, serious impairment of recognition and spatial memory was observed in PS cDKO mice at the age of 5 m (22). PS cDKO mice are an appropriate model widely accepted owing to the recapitulation of typical AD pathological features and deterioration process with the increase of age. Hence, behavioral tests were used to evaluate the memory function for determining whether GZFL was able to improve the impaired cognitive ability in PS cDKO mice. In agreement with previous studies, severe behavioral deficits were observed in PS cDKO mice, including recognition memory, spatial memory, and associative memory (16). Specifically, an open field experiment demonstrated that GZFL had no influences on the spontaneous locomotor ability of PS cDKO mice. To our surprise, administering GZFL to PS cDKO mice significantly improved recognition memory in a novel object recognition task. Moreover, a remarkable improvement of impaired spatial memory was discovered in PS cDKO mice treated with GZFL by implementing Y-maze and MWM. Particularly, PS cDKO mice receiving GZFL treatment performed significantly higher levels of contextual and cued freezing, indicating that GZFL had a great advantage in relieving the associative memory of PS cDKO mice. In summary, GZFL treatment led to an amelioration of cognitive deficits, which undoubtedly manifested the neuroprotective effects of GZFL on AD-like behaviors in PS cDKO mice.

Studies have found that the best correlates of cognitive impairment in AD patients are synapses injury and loss (34). NMDA receptors are ligand-gated ion channels, which play a pivotal role in formation of memory (35). As the main markers of synapses, SYP and PSD95 proteins play the role of basic scaffold proteins in the formation of synapses and neural development, which are closely related to the function of neurons and synapses (36). Moreover, cognitive decline is relevant to the impairment of SYP in HPC and other associated cortices (37). MAP2, a sensitive marker proved for age-related changes in rodents (38) and nonhuman primates (39), makes a significant contribution to stabilize microtubules (MT) and dendritic plasticity. Therefore, we further examined the effects of GZFL treatment on synaptic function in PS cDKO mice by detecting the expression changes of these associated synaptic protein in HPC. In agreement with previous studies, PS cDKO mice showed a dramatic reduction in the expressions of these five synaptic proteins (NR2A, NR2B, MAP2, SYP and PSD-95) in HPC compared to their littermates (16). Of note, GZFL treatment brought about an obvious increase in the expressions of synaptic proteins in HPC of PS cDKO mice. Together, these findings indicated that GZFL was of great assistance to PS cDKO mice for the improvement of the damaged synaptic function.

Tau is a MT-associated protein that concentrates on the stability of neuronal MT and regulation of axonal transport (40). As one of the earliest and continuous features during procession of AD, hyperphosphorylation of tau interrupts the association of tau with MT and causes tau to aggregate into oligomers, which is closely related with the onset of clinical symptoms (41). Pathological tau may do great damage to composition and structure of synapse, resulting in synaptic dysfunction and subsequent synapse loss (42). So far, a great deal of potential tau-targeting therapies has entered into the clinical trial stage for AD. However, most of them failed to translate into benefits for humans, most likely due to the poor efficacy or side effects (43). Phosphorylated tau at ser396 or ser404 sites served as a modified form can induce synaptic failure, generate intracellular deposits, and cause cognitive defects in AD (31). Since tau pathology is a complicated multifactorial process, the therapeutic strategy of traditional Chinese medicine compound with multiple targets might be promising. Thus, we investigated the phosphorylation of tau protein at ser396 and ser404 in HPC of PS cDKO mice after GZFL treatment. Our results indicated that the PS cDKO mice manifested significantly higher phosphorylation of tau at ser396 and ser404 in comparison with WT mice, which was consistent with previous findings (23). Strikingly, administering GZFL to mice gave rise to an obvious decrease in phosphorylation of tau in HPC of PS cDKO mice, which supported a possibility that the improvement of GZFL on memory impairment was decided by the inhibition of tau hyperphosphorylation.

Neuroinflammation, an essential part of initiation for AD pathology, is a process regulated by the microglia, which is capable of discriminating and eliminating any toxic components in the CNS (44). The activity of microglia has a great influence on synaptic pruning, maintenance of synaptic plasticity, neuronal apoptosis and immune monitoring, and it is synchronized with the development, maturation and aging of the CNS (45). Research has found that microglia in the aged brains have functional defect and are easy to be sustained activated, which might have a bearing on the pathogenesis of AD (46). In addition, clinical studies have found that cognitive performance is negatively correlated with microglia activation in individuals (47). Thus, a possible therapeutic way for AD is to suppress microglial activation. We observed the morphological changes of microglia in HPC of mice in each group by DAB immunostaining. Unsurprisingly, PS cDKO mice showed an increased proportion of amoeboid in total microglia with lower branching numbers, and both the average length and the longest branch length were shortened. Under pathological stimulation, highly differentiated microglia can be transformed into an amoeboid form (48), which is consistent with our findings. Microglia in the aging brain shrunk their area of surveillance due to the reduction of branches, resulting in the damage of homeostatic functions (49, 50). However, GZFL had the ability to inhibit the microglial activation, which could reverse these manifestations. In addition to the activation of microglia, the release of pro-inflammatory mediators is also a significant feature of chronic neuroinflammation (51). The sustained activation of microglia leads to the persistence of the inflammatory cycle, which further increases the levels of cytokines and chemokines (52). The excessive production of pro-inflammatory mediators makes microglia highly toxic under continuous conditions, so it is difficult for microglia to return to a resting state and eventually lead to neurodegeneration (53). Disturbances of inflammation in AD subjects are closely related to the changes of some acute-phase proteins and pro-inflammatory mediators in blood, cerebrospinal fluid and brain (52). Besides, a previous study has reported that the elevated levels of pro-inflammatory mediators such as TNF-α, IL-1β and IL-6 were found in the brain of AD patients (54). TNF-α mainly regulates cytokines activities by adjusting an autocrine cascade of production of IL-1 and TNF-α secreted from glial cells (55). IL-1β participates in the phosphorylation of tau and the synthesis of β-amyloid precursor protein (56). IL-6 could activate microglia, promote astrocyte proliferation and stimulate the production of acute-phase proteins (52). Our results showed a notable increase in the levels of IL-1β, TNF-α and IL-6 in HPC of PS cDKO mice, while GZFL treatment could decrease the levels of these pro-inflammatory mediators. It is worth noting that excessive accumulation of pro-inflammatory mediators such as COX-2 and iNOS endangers the survival of neurons in the brain (7). Furthermore, we found that the expressions levels of COX-2 and iNOS were higher in HPC of PS cDKO mice, and the increased expressions were reversed by GZFL administration. Given these benefits of GZFL, our research indicated that GZFL could play a key role in suppressing neuroinflammatory responses probably by inhibition of microglial activation in HPC of PS cDKO mice.

The significant effects of GZFL on memory impairment and inhibition of neuroinflammation in PS cDKO mice have been observed, however, the mechanism underlying how GZFL made contributions to neuroprotection remained unclear. Thus, we isolated and cultured primary microglia from neonatal rat cortex to further clarify the impacts and related mechanism of GZFL. As a potent activator of the immune system, LPS is capable of effectively inducing inflammatory responses by raising the release of NO, TNF-α, IL-6, and IL-1β in microglia (57). Thus, LPS is commonly used to study neuroinflammation in various studies. In our study, we found that primary microglia stimulated by LPS were converted into amoeboid ones, which exhibited larger spherical cell bodies surrounded by retracted processes. To our surprise, pretreatment of GZFL had the ability to promote the proliferation of microglia and prevent the transformation from static microglia to activated ones. Besides, we revealed that LPS stimulation increased the production of NO, COX-2, iNOS, IL-1β and TNF-α in primary microglia. However, we discovered that LPS-induced NO productions in primary microglia were dose-dependently reduced by pretreatment of GZFL without cytotoxic effects. Although no obvious dose-dependent changes in COX-2 and iNOS protein levels were observed after pretreatment with GZFL (12.5-400 μg/ml), GZFL led to a significant reduction in the levels of COX-2, iNOS, IL-1β, TNF-α and IL-6 in LPS-stimulated primary microglia by various extent. Considering the possible system or accidental errors, further *in vivo* and *in vitro* studies are merited to screen, refine the administration concentration of GZFL and calculate the EC50. Together, these results provided a convincing proof that GZFL had a strong influence on inhibiting neuroinflammation, which might largely depend on the repression of microglial activation.

JAK2/STAT3 signaling pathway plays a key role in cell proliferation, division, migration, tumor formation and immune cell maintenance (58). Accumulating evidences indicated that JAK2/STAT3 signaling pathway is a critical pathway that involves in inflammatory responses in the CNS (59). In order to explore the anti-inflammatory mechanism of GZFL, we detected the regulatory effect of GZFL on JAK2/STAT3 activation. In the present study, LPS stimulation for 6 h resulted in a dramatic increase in the expressions of phosphor-JAK2 and phosphor-STAT3 in primary microglia. However, the phosphorylation of JAK2 and STAT3 in LPS-stimulated primary microglia exposed to GZFL pretreatmen for 6 to 24 h was significantly reduced. Hence, these findings illustrated that GZFL specifically interfered with JAK2/STAT3 signaling pathway, which might be its potential mechanism in inhibiting microglial neuroinflammation. To pursue this speculation, we then used Ruxolitinib (an inhibitor of JAK2) and C188-9 (an inhibitor of STAT3) to further demonstrate the participation of JAK2/STAT3 pathway in the process of reducing inflammatory responses by GZFL treatment. Our results showed that the phosphorylations of JAK2 and STAT3 were obviously decreased by Ruxolitinib and C188-9, and the expressions of COX-2 and iNOS were evidently attenuated with the intervention of Ruxolitinib. These results from Ruxolitinib were consistent with the treatment of GZFL on anti-inflammatory effects in primary microglia stimulated by LPS. Importantly, combined treatment with GZFL and the two inhibitors had similar impacts as GZFL or inhibitor used alone. Namely, these three interventions may share the same mechanism, JAK2/STAT3 pathway. Meanwhile, it is worth noting that IL-6 has been demonstrated to be the primary activator of JAK2/STAT3 signaling (60). Therefore, it might be possible that the effect of GZFL on JAK2/STAT3 pathway in LPS-stimulated primary microglia is partly mediated by IL-6, which needs further investigation. Also noteworthy is that Traditional Chinese Medicine is characterized by multi-component, multi-target and multi-pathway. Hence, future studies using reliable analytical methods are needed to clear and definite the specific components of GZFL under our experimental conditions to further facilitate the understanding of the anti-AD effect of GZFL. Altogether, these results supported that GZFL improved neuroinflammatory responses by blocking the JAK2/STAT3 signaling pathway in LPS-stimulated primary microglia.

## Conclusions

**Figure 10:**
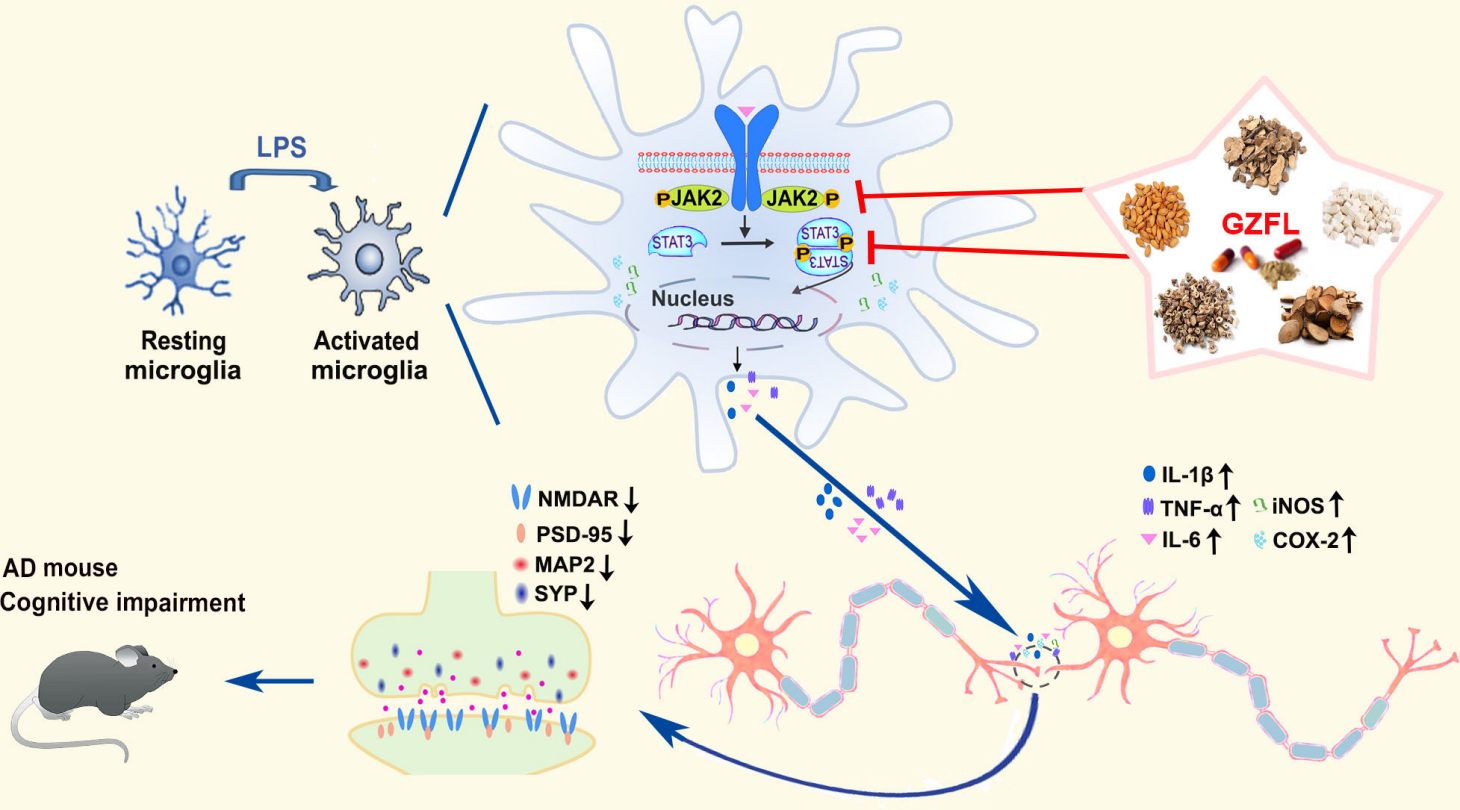
Summary diagram representing the effects of Guizhi Fuling capsule (GZFL) on improving memory deficits in presenilin1/2 conditional double knockout (PS cDKO) mice by inhibiting microglial neuroinflammation through blockage of JAK2/STAT3 signaling pathway.

As summarized in **Figure 10**, our findings supply hard evidence that GZFL has shown promising therapeutic effects on memory impairment and synaptic dysfunction in AD, which are due to the inhibition of microglial activation and neuroinflammation *via* obstruction of JAK2/STAT3 signaling pathway. Thus, as a classical Chinese herbal formula, GZFL may be a representative of potential anti-neuroinflammatory agent for ameliorating memory deficits and postponing the progression of neurodegenerative disorders that are intimately related to neuroinflammation.

## Data availability statement

The original contributions presented in the study are included in the article/**Supplementary Material**. Further inquiries can be directed to the corresponding authors.

## Ethics statement

The animal study was reviewed and approved by Animal Care and Use Committee of Shanghai University of Traditional Chinese Medicine.

## Author contributions

YX, JY and YG conceived and designed the research. YG, GY, YT, XW, CZ, ZB, RA, XL, WG, YZ performed the research. YX, JY and YG analyzed the data and prepared the manuscript. All authors contributed to the article and approved the submitted version.
