# Supplementary figures and images for: Guizhi Fuling capsule relieves memory deficits by inhibition of microglial neuroinflammation through blocking JAK2/STAT3 pathway in presenilin1/2 conditional double knockout mice
Source: Front Immunol. 2023 Jul 3;14:1185570. doi: 10.3389/fimmu.2023.1185570 (PMC10350565; doi:10.3389/fimmu.2023.1185570)

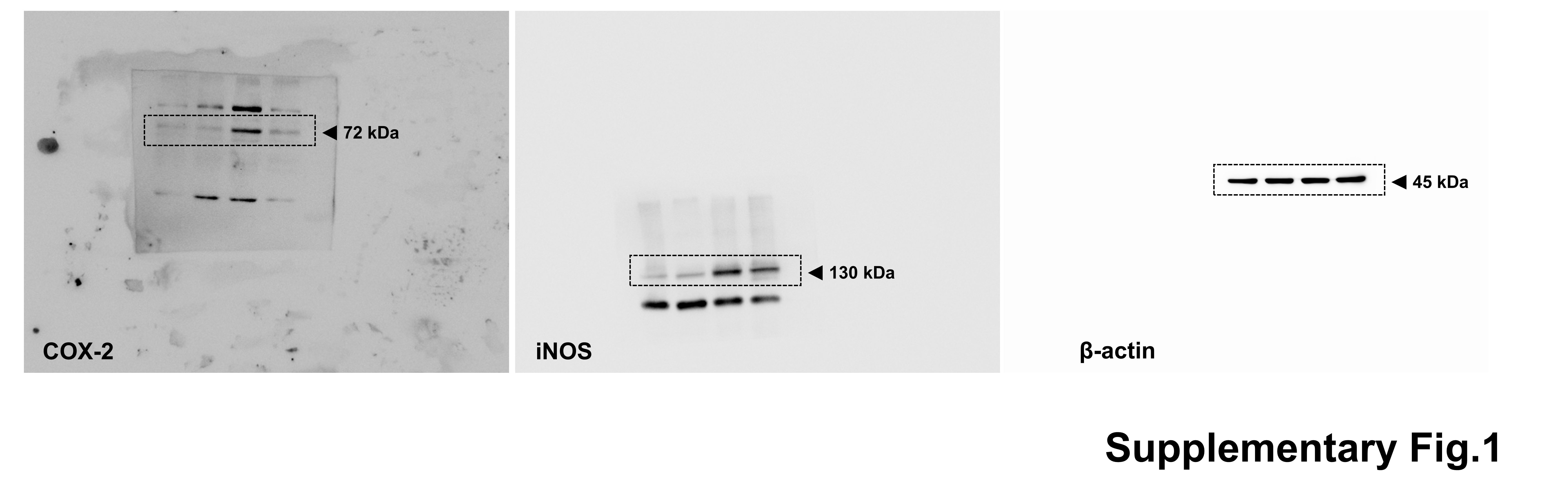

Supplement: Supplementary file 1 [file Image_1.tif]

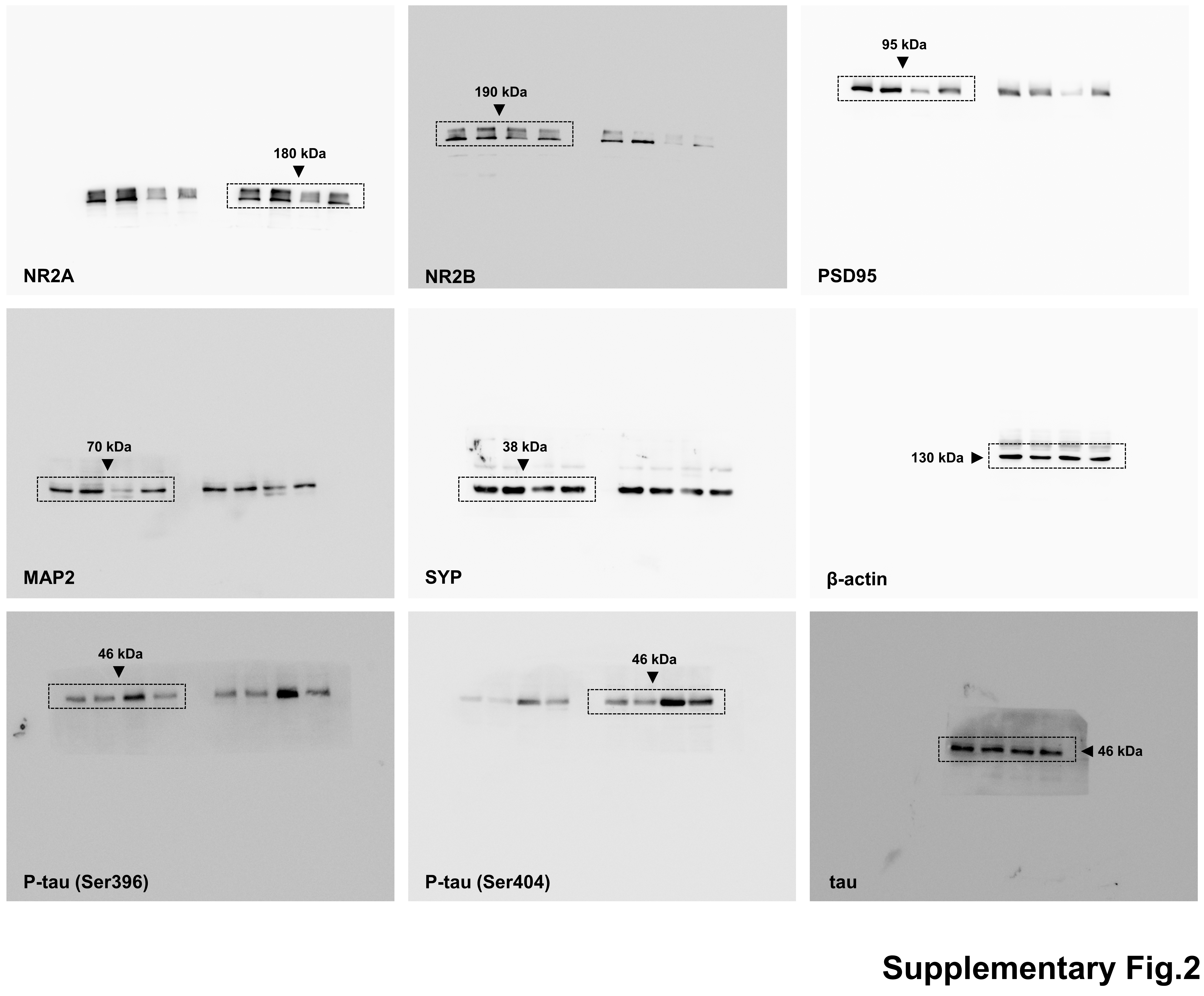

Supplement: Supplementary file 2 [file Image_2.tif]

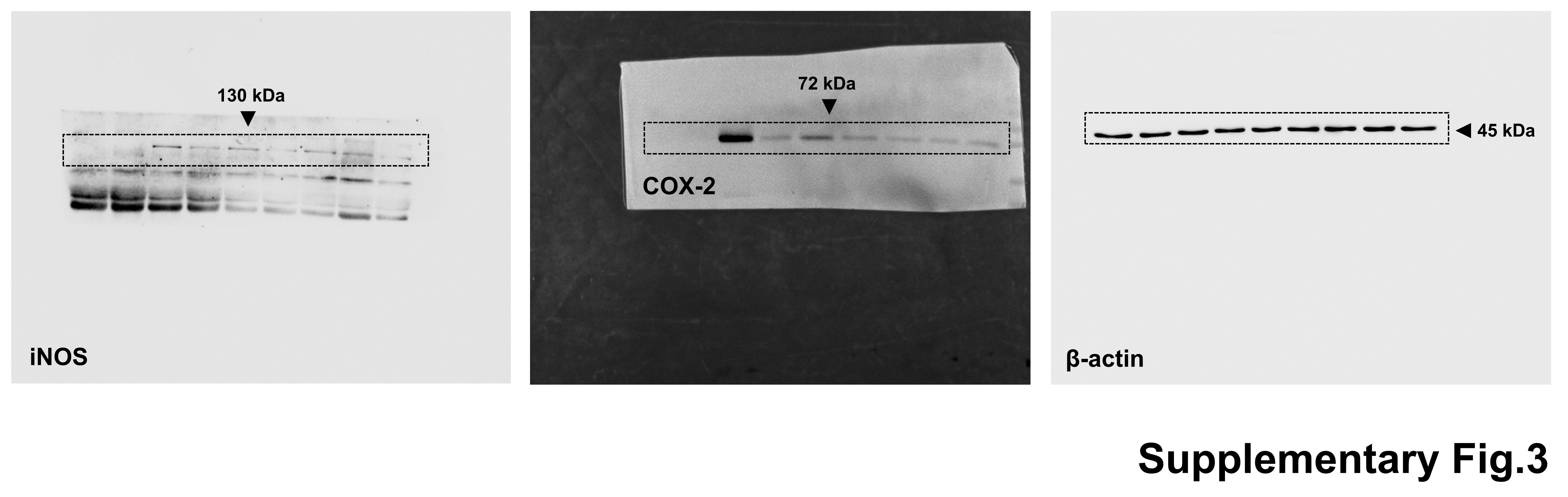

Supplement: Supplementary file 3 [file Image_3.tif]

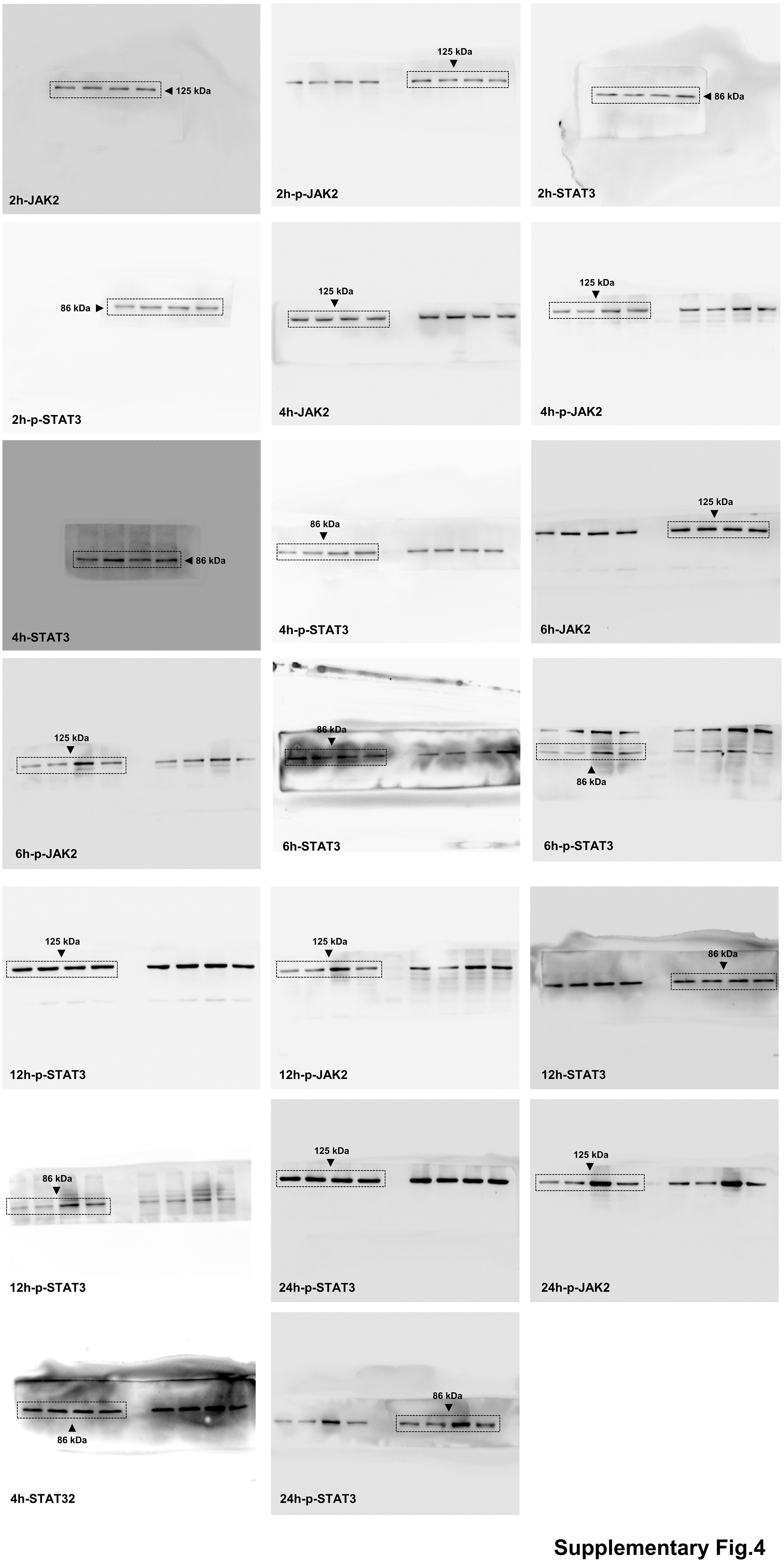

Supplement: Supplementary file 4 [file Image_4.tif]

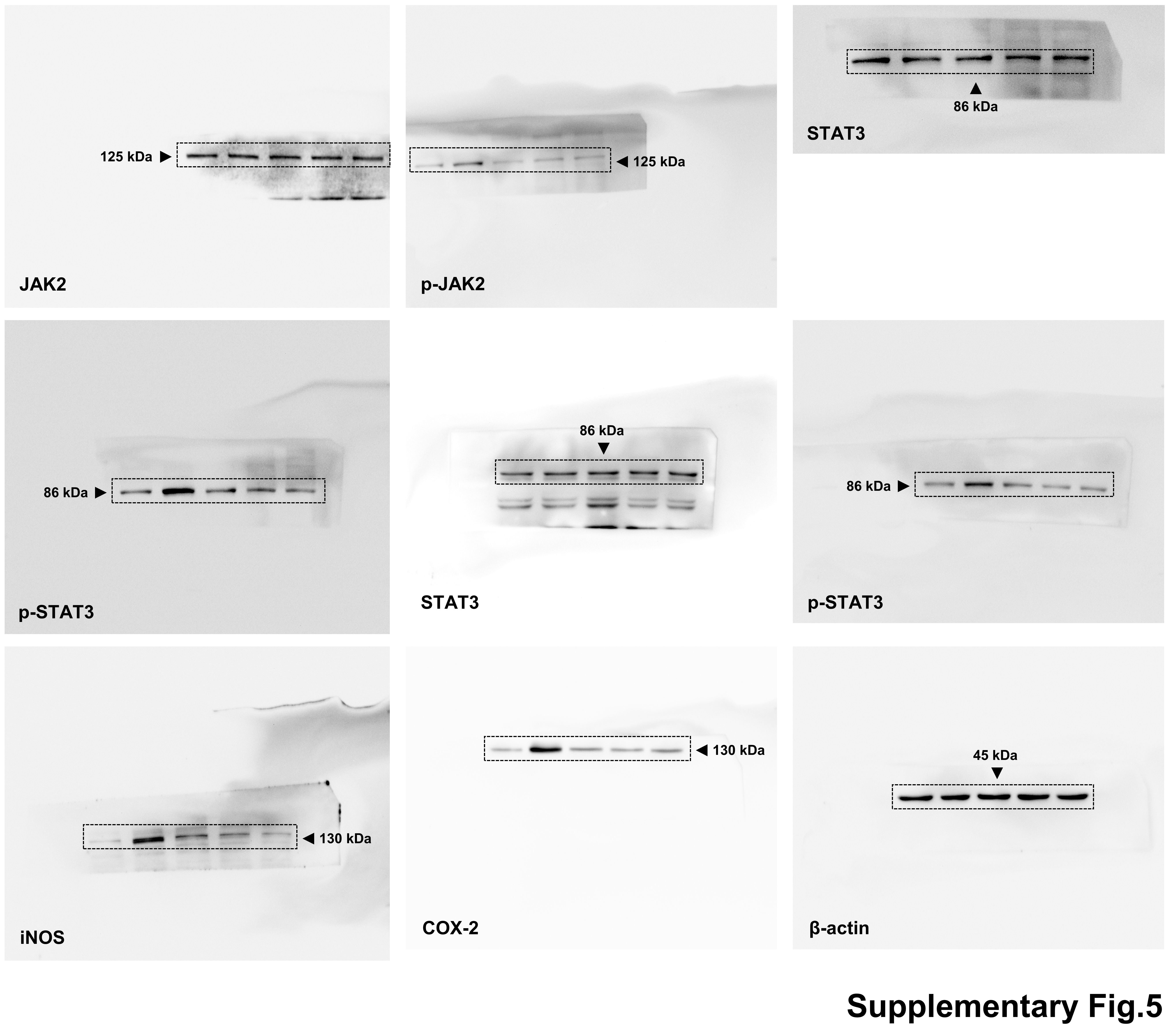

Supplement: Supplementary file 5 [file Image_5.tif]
