# Supplementary material for: Guizhi Fuling capsule relieves memory deficits by inhibition of microglial neuroinflammation through blocking JAK2/STAT3 pathway in presenilin1/2 conditional double knockout mice
Source: Front Immunol. 2023 Jul 3;14:1185570. doi: 10.3389/fimmu.2023.1185570 (PMC10350565; doi:10.3389/fimmu.2023.1185570)
Supplement: Supplementary file 6 [file Table_1.docx]

**Supplemental information**

Table S1. The primer sequences of COX-2, iNOS, TNF-α, IL-1β, IL-6, β-actin

| Gene | Forward (5’-3’) | Reverse (5’-3’) |
| --- | --- | --- |
| COX-2 | gtctggtgcctggtctgatga | tggtaaccgctcaggtgttg |
| iNOS | caccttggagttcacccagt | accactcgtacttgggatgc |
| TNF-α | gaactggcagaagagaggcact | agggtctgggccatagaact |
| IL-1β | caggcaggcagtatcactca | agctcatatgggtccgacag |
| IL-6 | ccacttcacaagtcggaggctta | gcaagtgcatcatcgttgttcatac |
| β-actin | agccatgtacgtagccatcc | tctcagctgtggtggtgaag |
